# Characterization of the complete mitochondrial genome of a holothurians species: *Holothuria hilla* (Holothuroidea: Holothuriidae)

**DOI:** 10.1080/23802359.2019.1660267

**Published:** 2019-09-03

**Authors:** Qiuhua Yang, Qi Lin, Fuyuan Yang, Jianshao Wu, Zhen Lu, Shengkang Li, Chen Zhou

**Affiliations:** a Key Laboratory of Cultivation and High-Value Utilization of Marine Organisms in Fujian Province, Fisheries Research Institute of Fujian, Xiamen, China;; b Guangdong Provincial Key Laboratory of Marine Biology, Marine Biology Institute, Shantou University, Shantou, China

**Keywords:** *Holothuria hilla*, mitochondrial genome, phylogenetic analysis

## Abstract

Mitochondrial genome sequence is a great potential method to both resolve disputed taxonomic issues and to infer phylogenetic relationships among holothurians. In this study, we present the complete mitochondrial genome of *Holothuria hilla* which was 15,744 bp in length, containing 13 protein-coding genes, 2 rRNA genes, 22 tRNA genes, and a putative control region. The gene content and arrangement were typical for Holothuroidea ground pattern. The overall base composition was 32.43% A, 27.20% T, 24.35% C and 16.02% G, showing a bias toward A + T (59.63%). The maximum-likelihood tree based on the concatenated 13 protein-coding genes revealed the phylogenetic relationships among the Holothuroidea species.

The holothurians *Holothuria hilla*, belongs to the Holothuroidea, Aspidochirotida, Holothuriidae, *Holothuria*, *Thymiosycia* (Liao 1997), is widely distributed in most countries of the western central Pacific to Pitcairn Islands and found in Southeast Asia, east Africa, and Indian Ocean (Purcell et al. 2012). Muscle sample of a wild *H. hilla* individual was collected from Changjiang, Hainan Province of China (19°26′51ʺN, 108°51′56ʺE). The specimen was preserved in the Culture Collection of Sea Cucumber at the Fisheries Research Institute of Fujian of China (specimen number: 2018092521) and stored at −80 °C for DNA isolation. Genomic DNA extraction, PCR amplification, sequencing, and annotation were performed according to the methods described by Yang et al. (2015).

The complete mitogenome of *H. hilla* (GenBank accession no. MN163001) is a circular DNA molecule with a length of 15,744 bp, which composed of 13 protein-coding (PCGs), 2 ribosomal RNA (rRNA), 22 transfer RNA (tRNA) genes, and 1 control region. All the genes of *H. hilla* encoded on the H-stand with the exception of one PCG (*nad6*) and eight tRNAs (*tRNA-Ser*, *tRNA-Gln*, *tRNA-Ala*, *tRNA-Val* and *tRNA-Asp*), which was typical for Holothuroidea mitogenomes (Uthicke et al. 2009; Perseke et al. 2010; Xia et al. 2016). Overall, nucleotide base composition of *H. hilla* mitogenome was calculated by MEGA 6 (MEGA Inc., Tampa, FL, USA) (Tamura et al. 2011): 32.43% A, 27.20% T, 24.35% C, and 16.02% G with an overall A + T content of 59.63%. The 13 protein-coding genes encode 3778 amino acids in total. Most of the protein-coding genes use the initiation codon ATG, except *nad4L* uses GTG. The most frequent stop condons were TAA, except *nad4* and *nad6* is terminated with TAG and *cox2* with an incomplete stop codon T.

The *12S* rRNA and *16S* rRNA were 825 bp and 1558 bp in length, respectively, and located in the typical position (Mu et al. 2018). The 22 tRNA genes were interspersed between rRNAs and protein-coding genes, with sizes ranging from 60 bp (*tRNA-Tyr*) to 72 bp (*tRNA-Leu*^CUN^). All 22 tRNA genes were predicted to be capable of folding into a clover-leaf secondary structure using tRNAscan-SE 1.21 (Lowe and Eddy 1997). The control region was located between *tRNA-Thr* and *tRNA-Pro* genes with 403 bp in length, with a higher A + T content (63.03%). All the features mentioned above are similar with the typical Holothuroidea mitogenome (Shen et al. 2009; Fan et al. 2012).

To determine the taxonomic status of *H. hilla*, we performed the phylogenetic analysis on the basis of the concatenated 13 protein-coding genes using maximum-likelihood method (Swofford 2002), using two Ophiuroidea species as outgroup species (Figure 1). We performed bootstrap analyses (1000 replicates) for both weighting schemes to evaluate relative levels of support for various nodes in the phylogenies. The phylogenetic tree clearly demonstrated that *H. hilla*, as well as the other Holothuriidae species clustered in a clade and formed a sister-relationship with Stichopodidae.

**Figure 1.**
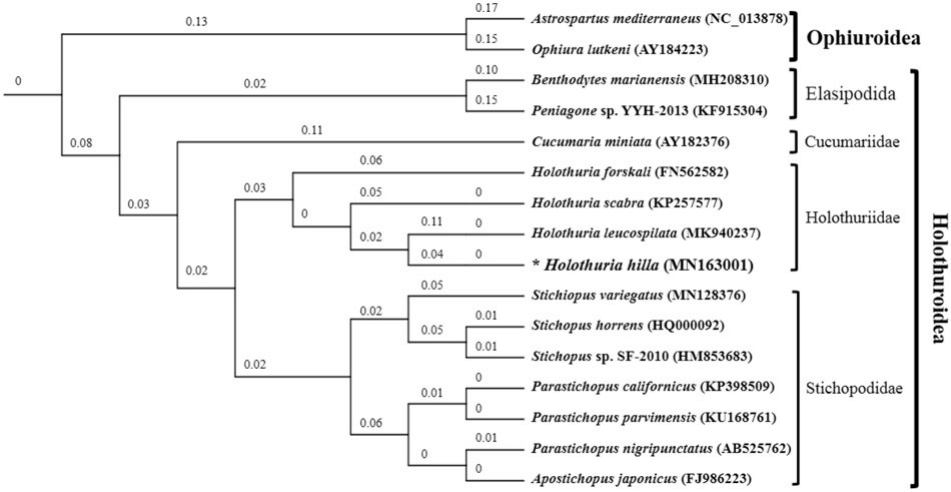
Maximum-likelihood tree inferred from 13 protein-coding genes of mitochondrial genomes of 14 Holothuroidea species, using two Ophiuroidea species as outgroups. The bootstrap values are based on 1000 re-samplings. The number at each node is the bootstrap probability. The number after the species name in the brackets is the GenBank accession number. The asterisks before species names indicate newly determined mitochondrial genome in this paper.
